# Seasonality and changing prevalence of common canine gastrointestinal nematodes in the USA

**DOI:** 10.1186/s13071-019-3701-7

**Published:** 2019-09-05

**Authors:** Jason Drake, Tom Carey

**Affiliations:** Elanco Animal Health, 2500 Innovation Way, Greenfield, IN 46140 USA

**Keywords:** *Toxocara*, *Ancylostoma*, *Trichuris*, Roundworm, Hookworm, Whipworm, Dogs, USA

## Abstract

**Background:**

The three most commonly diagnosed gastrointestinal parasites of pet dogs within the USA are the whipworm, the hookworm, and the roundworm. The collection of large data sets from various sources throughout the industry have produced a number of publications on parasite prevalence in recent years. In this study, we look at data captured by the Companion Animal Parasite Council from 2012–2018, which includes 4.3–7.2 million annual fecal exams, to evaluate not only changes in prevalence, but also possible seasonal fluctuations of the three most common canine gastrointestinal parasites.

**Methods:**

Annual and monthly data were collected from the CAPC parasite prevalence maps for canine roundworms, hookworms and whipworm. The map data were provided to CAPC by two large national reference laboratories. The data were evaluated for changes in prevalence on a monthly basis throughout each year as well as changes in prevalence from year to year from 2012–2018. Additionally, positive test results and total tests performed for each of the three parasites from 2012–2018 during individual months were totaled without using the year as a variable in order to evaluate the results for seasonality (i.e. all tests and positive results occurring in January, regardless of year, were totaled and analyzed).

**Results:**

Evaluation of gastrointestinal nematode prevalence data from over 39 million fecal samples examined over a 7-year period revealed a subtle, yet significant, increasing prevalence for roundworms, an increasing prevalence for hookworms, and a slightly decreasing prevalence for whipworms. Seasonality was demonstrated for roundworms, hookworms, and to our knowledge, for the first time canine whipworms. Highest seasonal prevalence for roundworms, hookworms, and whipworms occurred during December–January, July–August, and January–February, respectively.

**Conclusions:**

Evaluation of monthly gastrointestinal parasite prevalence data from over 39 million fecal samples collected over a 7-year period revealed a slightly increasing prevalence for roundworms, an increasing prevalence for hookworms, and a slightly decreasing prevalence for whipworms. In addition to the annual changes in prevalence, seasonal prevalence was shown for the first time for whipworms. Prevalence of both whipworm and roundworm peaked in the winter, while prevalence of hookworm peaked in the late summer and early autumn.

## Background

In the USA, the three most prevalent gastrointestinal parasites of dogs have been the whipworm *Trichuris vulpis*, the hookworm *Ancylostoma caninum*, and the roundworm *Toxocara canis* [1–7]. When compared to roundworm and hookworm, diagnosis of whipworm infection can be challenging for veterinary practitioners for a number of reasons. Whipworms exhibit intermittent egg shedding, relatively low numbers of eggs produced per worm compared to other common gastrointestinal nematodes like canine roundworm (*T. canis*) and canine hookworm (*A. caninum*), and production of very dense eggs that are difficult to find using passive fecal floatation with solutions of specific gravity less than 1.027 [8, 9]. The recent availability of fecal antigen diagnostic tests, which can detect roundworm, hookworm and whipworm infections in dogs, should improve the ability of veterinarians to diagnose infections with these gastrointestinal nematodes [10, 11].

The prevalence of gastrointestinal nematodes in dogs in the USA has been the topic of many publications in recent years. Historically, several parasite prevalence studies were reported by universities with the ability to evaluate hundreds or thousands of samples over several years [1–4]. More recently, larger parasite prevalence datasets have become available from corporate veterinary hospital groups and diagnostic laboratories with national or international clientele, allowing analyses and publication of national parasite prevalence data utilizing test results from hundreds of thousands or even millions of veterinary patients [5–7]. National studies of gastrointestinal nematode prevalence in pet dogs receiving veterinary care indicate a prevalence of roundworms of 1.8–5.0% [5–7], hookworms of 2.5–4.5% [5, 6] and whipworms of 0.8–1.2% [5, 6].

Seasonality of roundworm infections in dogs, with peak prevalence during the winter and lowest prevalence during the summer every year, has been previously described within other studies of canine parasite prevalence [1–3, 12, 13]. Seasonality of hookworm infections in dogs, with peak prevalence in the summer and autumn has also been published within results of other studies and is consistent with the biology of hookworm infections, where the infective larvae are sensitive to cold weather, thus limiting infections to times with warm, humid conditions adequate for sustainment of larvae [1, 2, 13–15]. Seasonality of *Trichuris* spp., while previously described in sheep and in pigs [16, 17], to our knowledge has not been previously described for *Trichuris vulpis* in dogs.

The Companion Animal Parasite Council (CAPC) produces parasite prevalence maps utilizing combined data provided by two large national reference laboratories [18]. Recent changes to these CAPC parasite prevalence maps allow access to monthly and yearly data on a county, state and national level, enabling in-depth evaluation of seasonality and changes in prevalence over several years and across large geographies as they include annual fecal exam results between 4.3–7.2 million dogs per year between 2012–2018 [18]. In this study, changes in prevalence and seasonal fluctuations in canine roundworms, whipworms and hookworms from 2012 to 2018 were analyzed.

## Methods

Annual and monthly data were collected from the CAPC parasite prevalence maps for canine roundworms, hookworms and whipworm. The data were evaluated for changes in prevalence on a monthly basis throughout each year as well as changes in prevalence from year to year between 2012–2018.

In order to evaluate the statistical significance of changes in incidence compared to 2012, 2 × 2 contingency tables of positive and negative results for each year were constructed and the data were assessed through Chi-square tests in order to determine if there were trends in annual prevalence for any of the three nematodes. Additionally, positive test results and total tests performed for each of the three parasites between 2012–2018 during individual months were totaled without using the year as a variable in order to evaluate the results for seasonality (i.e. all tests and positive results occurring in January, regardless of year, were totaled and analyzed.) This was conducted for all months. The two months with the highest and lowest overall prevalence were determined for each of the three parasites and analyzed through 2 × 2 contingency tables through Chi-square tests. This method was considered the most appropriate approach based upon available data; however, two possible weaknesses should be highlighted. First, this method assumes all data points are mutually statistically independent. Based on testing guidelines, it is likely that some dogs within the dataset will be represented more than once. It is not possible, however, to calculate the degree of correlation between observations because the data are only available at the monthly and yearly summary level and individual dog identity is not included. Secondly, the very large sample size means the Chi-square test can detect small differences. Some of these differences could be small and potentially inconsequential yet detected and declared significant. Therefore, both the significance and clinical relevance need to be assessed in order to provide full context.

## Results

### Annual changes in prevalence

**Fig. 1:**
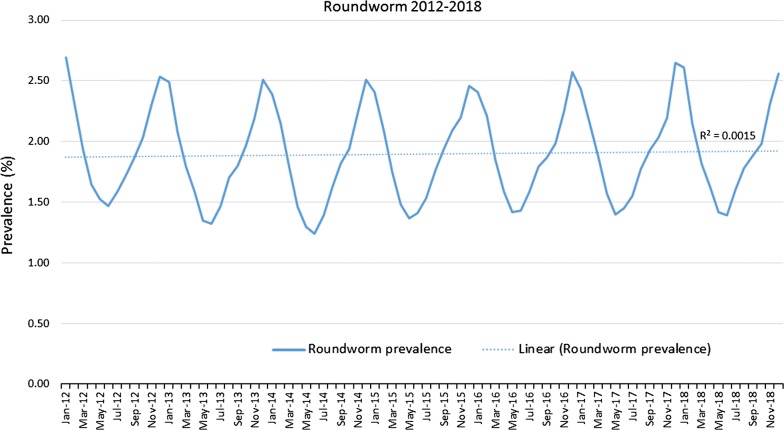
Roundworm prevalence 2012–2018

**Fig. 2:**
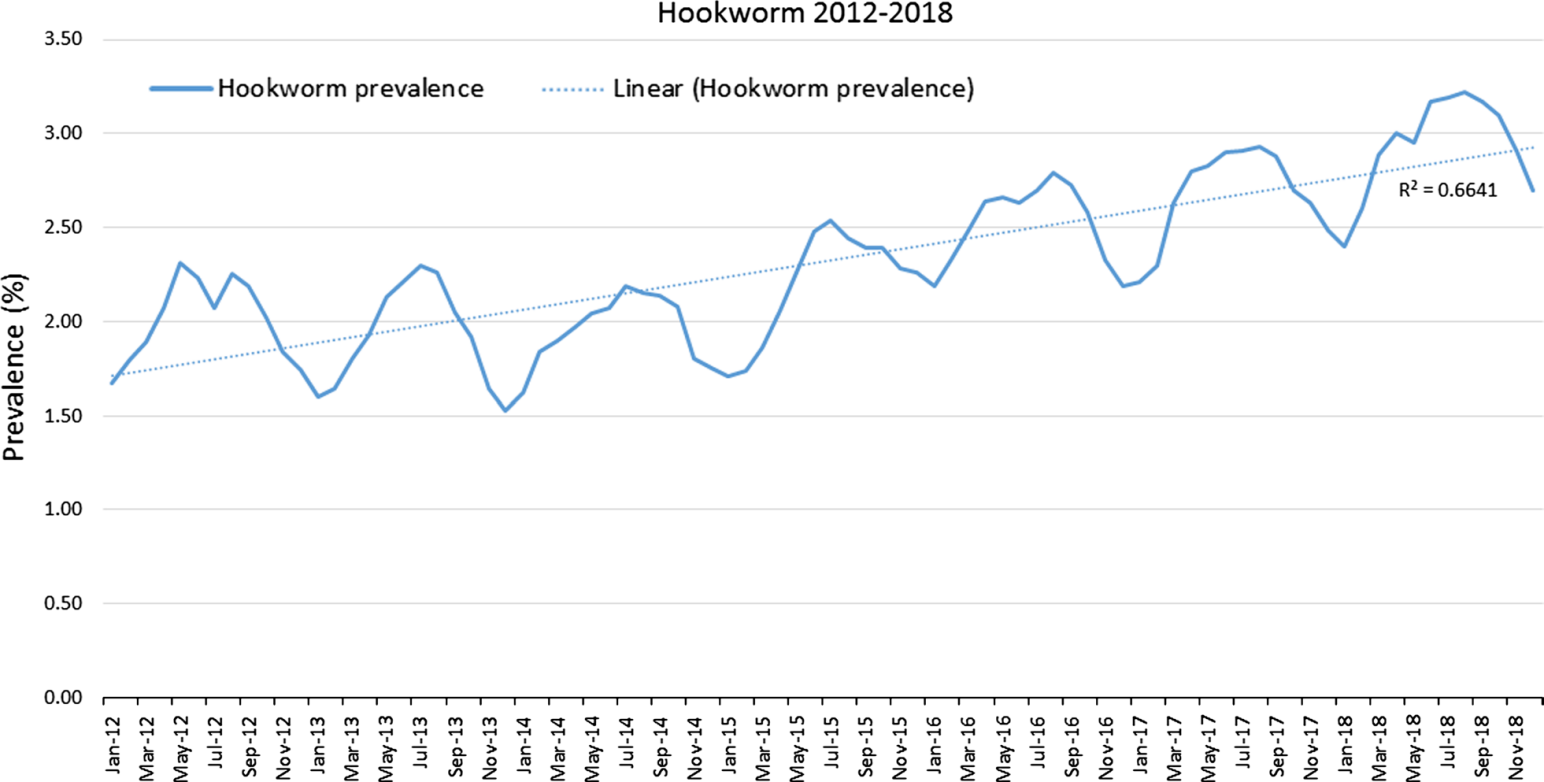
Hookworm prevalence 2012–2018

**Fig. 3:**
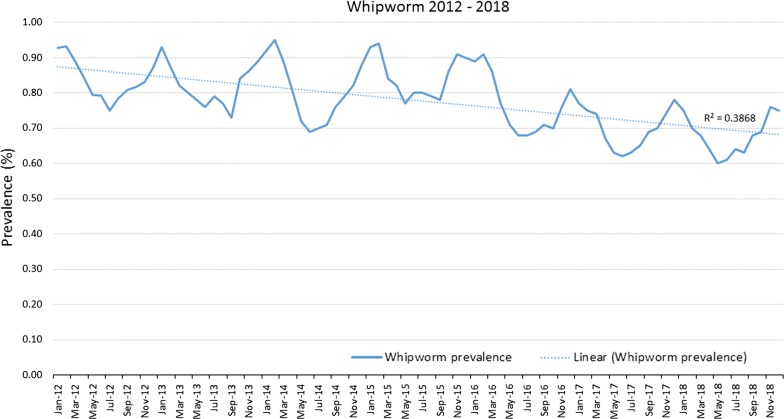
Whipworm prevalence 2012–2018

Evaluation of gastrointestinal nematode prevalence data from over 39 million fecal samples examined over a 7-year period revealed a subtle fluctuation in prevalence for roundworms (*P* < 0.0001) (Fig. 1), an increasing prevalence for hookworms (*P* < 0.0001) (Fig. 2), and a slightly decreasing prevalence for whipworms (*P* < 0.0001) (Fig. 3). Yearly prevalence for canine roundworms from 2012 to 2018 fluctuated slightly, falling from 1.94% in 2012 down to 1.77% in 2014, then rising to 1.89% in 2018. (Table 1). Yearly prevalence for canine hookworms from 2012 to 2018 also increased, starting at 2.02% in 2012 and reaching 2.96% by 2018 (Table 1). Yearly prevalence of canine whipworms decreased slightly, starting at 0.83% in 2012 and gradually dropping to 0.67% by 2018 (Table 1).

**Table 1:** Yearly prevalence

Year	Total samples	Roundworm positive (%)	Hookworm positive (%)	Whipworm positive (%)
2012	4,327,474	83,802 (1.94)	87,484 (2.02)	36,120 (0.83)
2013	4,652,951	84,284 (1.81)	90,449 (1.94)	37,946 (0.82)
2014	5,016,643	88,950 (1.77)	99,195 (1.98)	39,996 (0.8)
2015	5,515,328	101,278 (1.84)	122,372 (2.22)	46,262 (0.84)
2016	6,164,315	115,844 (1.88)	156,291 (2.54)	46,868 (0.76)
2017	6,777,847	127,732 (1.88)	182,955 (2.7)	46,964 (0.69)
2018	7,187,002	125,835 (1.89)	212,860 (2.96)	48,300 (0.67)

### Monthly changes in prevalence

**Fig. 4:**
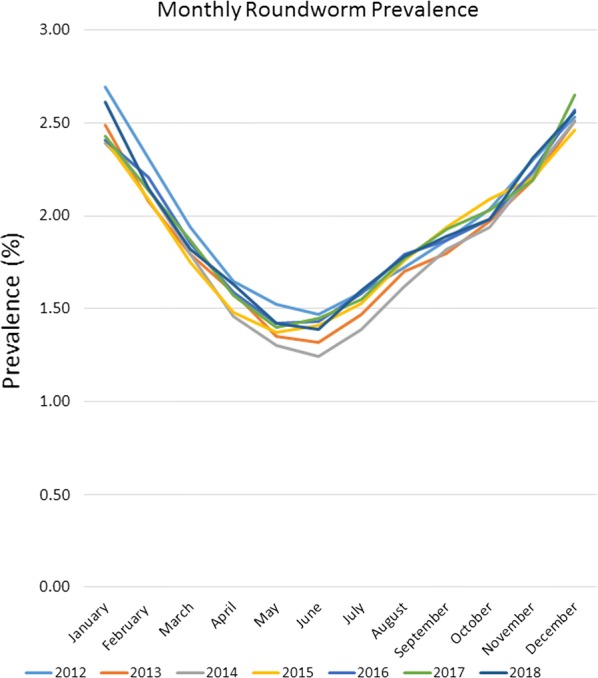
Monthly roundworm prevalence 2012–2018

**Fig. 5:**
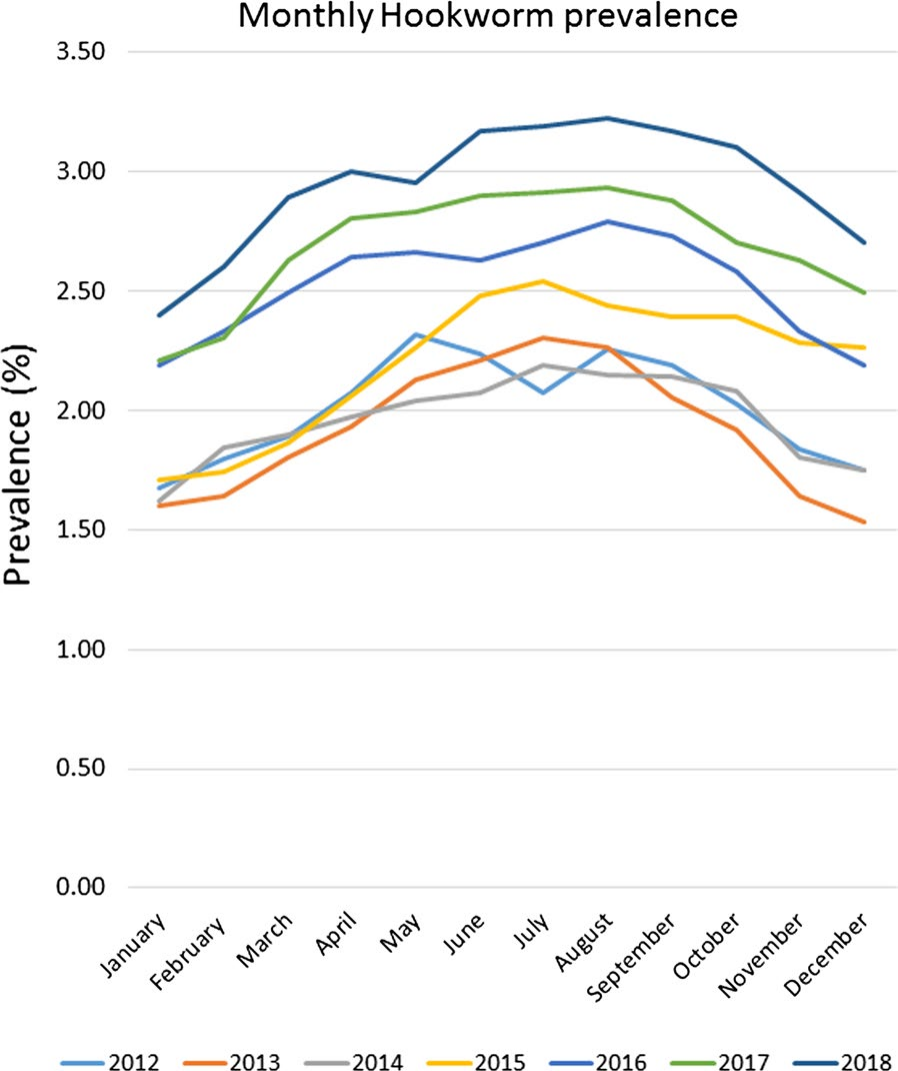
Monthly hookworm prevalence 2012–2018

**Fig. 6:**
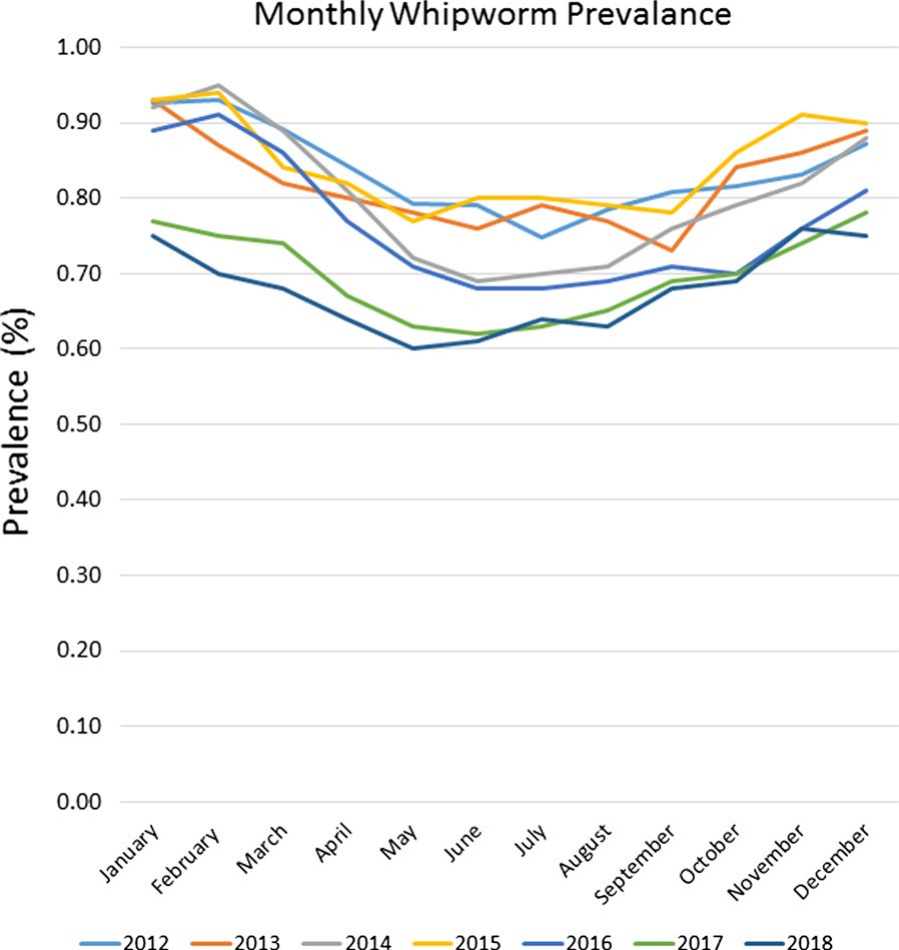
Monthly whipworm prevalence 2012–2018

Seasonality was demonstrated for roundworms, hookworms, and to our knowledge, for the first time canine whipworms. Monthly roundworm prevalence was consistent each year, with highest seasonal prevalence of 2.39–2.70% occurring during December-January and lowest prevalence of 1.24–1.52% occurring during May-June (*P* < 0.0001) (Fig. 4). The highest monthly hookworm prevalence in 2012 was 2.31% in May 2012, rising over the 7-year period to a peak summer prevalence of 3.22% in August 2018. Each year from 2013 onward, hookworm prevalence was highest in mid-summer and at the lowest in the winter. Hookworm prevalence was 2.08–3.22% during July–August and 1.60–2.60% during January-February (*P* < 0.0001) (Fig. 5). With a seasonal pattern similar to roundworm, the highest monthly whipworm prevalence of 0.70–0.95% during winter months of January-February and lowest monthly prevalence of 0.60–0.80% during May-June (*P* < 0.0001) (Fig. 6).

## Discussion

Overall, annual canine gastrointestinal parasite prevalence was similar to historic levels over the 7-year period evaluated. Monthly prevalence and annual trends revealed some very interesting insights. Yearly prevalence of roundworms was consistent, with a subtle fluctuation from year to year. Yearly prevalence of whipworms decreased slightly, with prevalence remaining above 0.8% from 2012 through 2015, declining to a prevalence of 0.67% in 2018, representing a 16% reduction in prevalence from 2012 to 2018 (*P* < 0.0001). While roundworm prevalence remained steady and whipworm prevalence slightly decreased, yearly hookworm prevalence increased nearly every year, starting at 2.02% in 2012 and increasing to 2.96% by 2018, representing a 47% increase in yearly prevalence from 2012 to 2018 (*P* < 0.0001). While additional research is needed to determine the causes of the slight decreasing whipworm prevalence and the increasing hookworm prevalence, a recent study identified the emergence of multi-drug resistant *A. caninum* hookworms [19]. Since the prevalence and geographical location(s) of multi-drug resistant hookworms have yet to be fully described we are unable to attribute the changes in prevalence to resistance at this time. Additional surveillance related to hookworms and drug sensitivity should be performed. Additionally, since hookworm larvae are sensitive to environmental factors, changes in climate or local weather fluctuations could also potentially have an impact on hookworm prevalence in areas where these changes favor improved larval survival [20].

Seasonality of roundworm infections in dogs, with peak prevalence during the winter and lowest prevalence during the summer every year, was consistent with previously published findings from other studies of canine parasite prevalence [1–3, 13, 14]. Seasonality of *T. canis* could be related to evolutionary canid reproduction cycles and parasite/host co-evolution. Some of the seasonality could also be associated with changes in the population signalment each month, if certain times of year have higher populations of puppies than others. Since no signalment data are provided *via* the CAPC maps, additional research may be indicated. Seasonality of hookworm infections in dogs, with peak prevalence in the summer and autumn, also fits with the published results of other studies and the biology of hookworm infections, where the infective larvae are sensitive to cold weather, limiting infections to times with warm, humid conditions adequate for sustainment of larvae [1, 2, 13, 15, 16]. Seasonality of *Trichuris* spp. has been previously described in sheep and in pigs [17, 18], but this may be the first description of seasonality for *T. vulpis* in dogs. The peak of whipworm infections in dogs occurred in the winter. Whipworm eggs are not infective when passed by the host and require approximately one month for first-stage larval development in order to become infective [21]. Following ingestion by a dog, the pre-patent period is slightly less than three months [21]. This time for egg development in the environment, combined with the long prepatent period, could be one explanation for the seasonal peak prevalence for whipworms in the winter.

Since no data were available regarding the treatment history or signalment of the dogs from which fecal samples were examined, additional factors could be influencing the data. It is possible that the percentage of young dogs and puppies in the population could be higher at certain times of the year, influencing prevalence. It is also possible that seasonal usage of dewormers or heartworm prevention in some parts of the USA could be lowering prevalence during the warmer months of the year. Hookworm prevalence increases during the same time of year as peak heartworm transmission season, when it is warmer, despite the likelihood that at least a portion of these dogs is receiving heartworm prevention that also treats hookworm infection. Of the roughly 70 million dogs in the USA, fewer than 20 million receive even a single dose of heartworm prevention [22]. While the nematode prevalence changes may be impacted by heartworm prevention, it is unlikely enough dogs are on prevention to completely explain the seasonality in prevalence seen.

## Conclusions

Evaluation of monthly gastrointestinal parasite prevalence data from over 39 million fecal samples collected over a 7-year period revealed a slightly increasing prevalence for roundworms, an increasing prevalence for hookworms, and a slightly decreasing prevalence for whipworms.

In addition to the annual changes in prevalence, seasonal prevalence was shown for the first time for whipworms. Prevalence of both whipworm and roundworm peaked in the winter, while prevalence of hookworm peaked in the late summer and early autumn. With the seasonal prevalence of different canine gastrointestinal parasites peaking at different times of the year, veterinarians and pet owners should consider year-round parasite control strategies that incorporate deworming measures against the three most common gastrointestinal nematodes found in dogs: hookworm, whipworm and roundworm.

## Data Availability

The gastrointestinal parasite testing datasets analyzed during the present study are available in the CAPC Prevalence Maps, https://www.capcvet.org/maps.

## Abbreviation

CAPC: Companion Animal Parasite Council
